# In-vivo-validation of a cardiovascular risk prediction tool: the arriba-pro study

**DOI:** 10.1186/1471-2296-14-13

**Published:** 2013-01-22

**Authors:** Annette Diener, Salomé Celemín-Heinrich, Karl Wegscheider, Kai Kolpatzik, Katrin Tomaschko, Attila Altiner, Norbert Donner-Banzhoff, Jörg Haasenritter

**Affiliations:** 1 Institute of General Practice, Rostock University Medical Center, 18055, Rostock, Germany; 2 Department of General Practice/Family Medicine, Medical Faculty, Philipps University Marburg, 35043, Marburg, Germany; 3 Department of Medical Biometry and Epidemiology, University Medical Center Hamburg-Eppendorf, 20246, Hamburg, Germany; 4 Federal Association of the AOK, 10178, Berlin, Germany; 5 AOK Baden-Württemberg, 70191, Stuttgart, Germany

## Abstract

**Background:**

Calculation of individual risk is the cornerstone of effective cardiovascular prevention. **arriba** is a software to estimate the individual risk to suffer a cardiovascular event in 10 years. Prognosis and the absolute effects of pharmacological and lifestyle interventions help the patient make a well-informed decision. The risk calculation algorithm currently used in **arriba** is based on the Framingham risk algorithm calibrated to the German setting. The objective of this study is to evaluate and adapt the algorithm for the target population in primary care in Germany.

**Methods/design:**

**arriba**-pro will be conducted within the primary care scheme provided by a large health care insurer in Baden-Württemberg, Germany. Patients who are counseled with **arriba** by their general practitioners (GPs) will be included in the **arriba**-pro cohort. Exposure data from the consultation with **arriba** such as demographic data and risk factors will be recorded automatically by the practice software and transferred to the study centre. Information on relevant prescription drugs (effect modifiers) and cardiovascular events (outcomes) will be derived from administrative sources.

**Discussion:**

The study is unique in simulating a therapy naïve cohort, matching exactly research and application setting, using a robust administrative data base, and, finally, including patients with known cardiovascular disease who have been excluded from previous studies.

**Trial registration:**

The study is registered with Deutsches Register Klinischer Studien (DRKS00004633).

## Background

Cardiovascular disease (CVD) is the leading cause of death in industrialized and, increasingly, in developing countries [1]. In 2008, 17.1 million deaths worldwide were caused by cardiovascular disease, which amounts to 30% of all deaths [2]. For Germany, the respective numbers are 342,000 cardiovascular deaths per year (40% of all deaths) [3]. The prevention of cardiovascular diseases is thus an area with high priority.

Within a high-risk prevention paradigm, persons with elevated cardiovascular risk are identified and treated [4]. Here the focus has shifted from the diagnosis and treatment of single risk factors to a view focusing on global cardiovascular risk. On the basis of multivariate algorithms clinicians can now calculate an individual’s cardiovascular risk. Behavioral and pharmacological interventions are offered only to high risk individuals. This approach maximizes absolute risk reduction and efficiency compared to the treatment of individual risk factors [4-6].

 The American Framingham study, starting 1948 and still continuing, has been the first prospective study to identify risk factors contributing to cardiovascular events [7]. From this study the presumably best known and most widely used risk algorithm was derived which was subsequently validated for different populations [8,9]. Although these studies revealed that the Framingham algorithm resulted in an overestimation of the actual observed risk in populations different from the derivation cohort (lack of calibration), its discrimination ability has repeatedly been shown to be excellent [10].

The majority of consultations regarding cardiovascular disease treatment and prevention takes place in primary care [11]. Several tools, most of them based on the Framingham algorithm, are available to support practitioners calculating their patients’ cardiovascular risk. In Germany, **arriba** (http://www.arriba-hausarzt.de) has become the most popular support tool. It has been developed by the Departments of General Practice at Philipps University Marburg and Rostock University. The **arriba** calculator is based on the Framingham risk algorithm with calibration for the German setting. This is achieved by a constant multiplier derived from population cohorts [12]. 10-year absolute risk for cardiovascular events is calculated based on age, sex, blood pressure, lipids, smoking status, family history and diabetes control [13]. For secondary prevention, a simplified estimate is presented based on a prevention trial cohort [14]. Individual risk as well as effects of medication and behavioral change can easily be demonstrated as absolute risk reduction. The software can be freely downloaded by individual general practitioners (GPs).

**arriba** has been designed to be used intuitively. Demonstration of effects of single intervention and their combinations does not require sophisticated understanding of absolute and relative risk reduction. **arriba** thus supports shared decision-making between physician and patient as well as adherence to treatment plans [15,16]. **arriba** has been evaluated in a cluster randomized controlled trial, in which patient satisfaction, participation, and change in cardiovascular diseases risk status were investigated. It could be shown that patients randomized to the intervention group had a significant higher satisfaction with process and result than controls [17]. The acceptance by **arriba** among primary care physicians varied, depending on their personal motivation, their attitude towards shared decision-making in general and context variables such as time constraints [18].

Since 2004, primary care in Germany has increasingly been provided within gatekeeping schemes (Hausarztzentrierte Versorgung – HzV). In the German federal state Baden-Württemberg **arriba** has been implemented as part of the HzV of the Allgemeine Ortskrankenkasse Baden-Württemberg (AOK BW), the largest statutory non-profit health insurance in the federal state Baden-Württemberg, Germany [19]. Regular automated data transfer and consent by patients who participate in the HzV has provided the opportunity to establish a cohort to improve the **arriba** risk algorithm.

The study we are presenting here has as its objective the validation and adaptation of the **arriba** risk algorithm for future use in primary care. We will not only address primary prevention, but also prediction for patients with known cardiovascular disease and/or diabetes mellitus.

## Methods/design

**arriba**-pro is a prospective, longitudinal cohort study based on medical and administrative data provided by primary care practices and a large health insurance company. Attending patients who enrolled in the HzV AOK BW and are counseled with **arriba** by their GPs are eligible.

### Participants and recruitment

In the Land of Baden-Württemberg (BW), about 1 million members of the insurance company AOK BW are voluntarily enrolled in the HzV AOK BW. 3,500 out of 7,100 GPs in BW have registered to provide primary care services for these patients. For all GPs providing HzV services specially designed software for documentation and billing is available, including the **arriba** software. Every patient enrolled in the HzV-scheme for who **arriba** is used can automatically be included into the study after the approval of his/her GP.

### Exposure, outcomes and effect modifiers

Exposure variables will include demographic data and cardiovascular risk factors (see Table 1).

**Table 1:** Exposure variables

Demographics	Age/year of birth
	Gender
	Insurance number
Cardiovascular risk factors	Total Cholesterol mmol/L or mg/dL
	HDL-Cholesterol mmol/L or mg/dL
	Smoking yes/no
	Systolic blood pressure mm Hg
	Patient on blood pressure lowering medication yes/no
	Diabetes yes/no
	Diabetes control glycosylated hemoglobin
	Known atherosclerotic disease yes/no
	Family history for cardiac disease yes/no

Acute coronary syndrome (fatal/non-fatal) and acute stroke (fatal/non-fatal), excluding transient ischaemic attacks, will be the main outcome of the study. Secondary outcomes will be overall death, hospitalization for cardiovascular disease, any hospitalization, revascularization procedures (coronary and/or carotid vascular beds), and outpatients’ cardiology consultations. The occurrence of these will be determined from administrative data (see below). Drug treatment prescribed after recruitment into the study will be analyzed as potential confounder or effect modifier.

### Data collection

**Figure 1:**
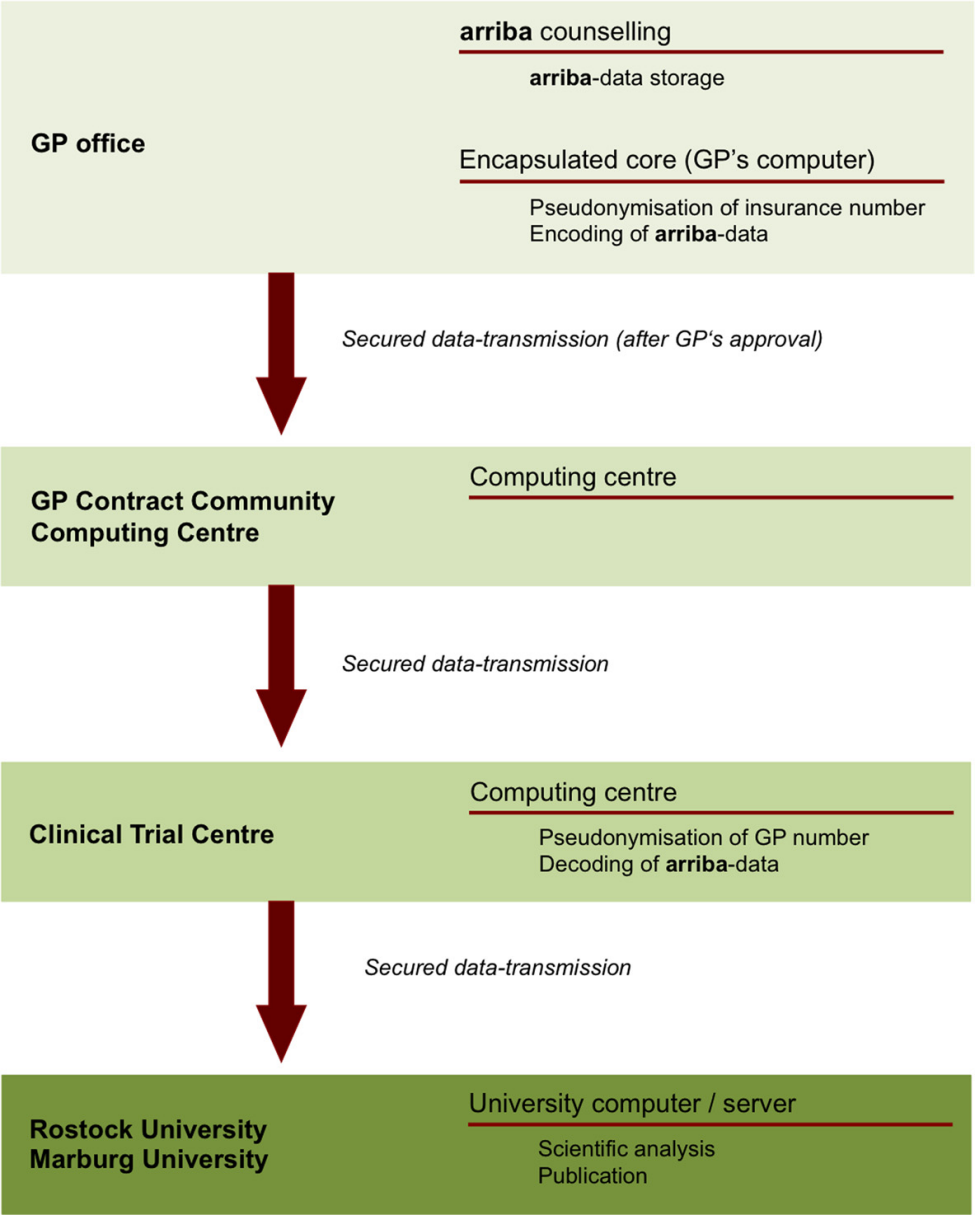
Data-transmission and pseudonymisation steps.

During a consultation supported by **arriba** GPs will enter the patient’s age, sex, cardiovascular risk factors and the interventions that have been discussed with the patient. These data together with the calculated absolute cardiovascular risk will automatically be stored within the **arriba** software. GPs will always be asked for their approval regarding each patient’s **arriba** data being stored by the software and transferred for further processing. Data will be regularly transferred to the central computing centre. From there the data will be sent to an independent clinical trial centre (CTC North at University Medical Center Hamburg Eppendorf). The CTC North will provide data files to the collaborating departments at the universities of Marburg and Rostock. These will include clinical data and pseudonymised identifiers for patients and GPs (see Figure 1).

Much care will be taken to preserve the confidentiality of data from **arriba** counselling. Patients ID-numbers will be pseudonymised with the key known only to AOK BW. Clinical data will be encrypted so that only the participating academic researchers will be able to read them. At the CTC North GPs identifiers will also be pseudonymised. The pseudonymisation procedure will allow to link information of the same patient from different data sources while also maintaining full confidentiality and privacy of patients’ and GPs’ data.

**Figure 2:**
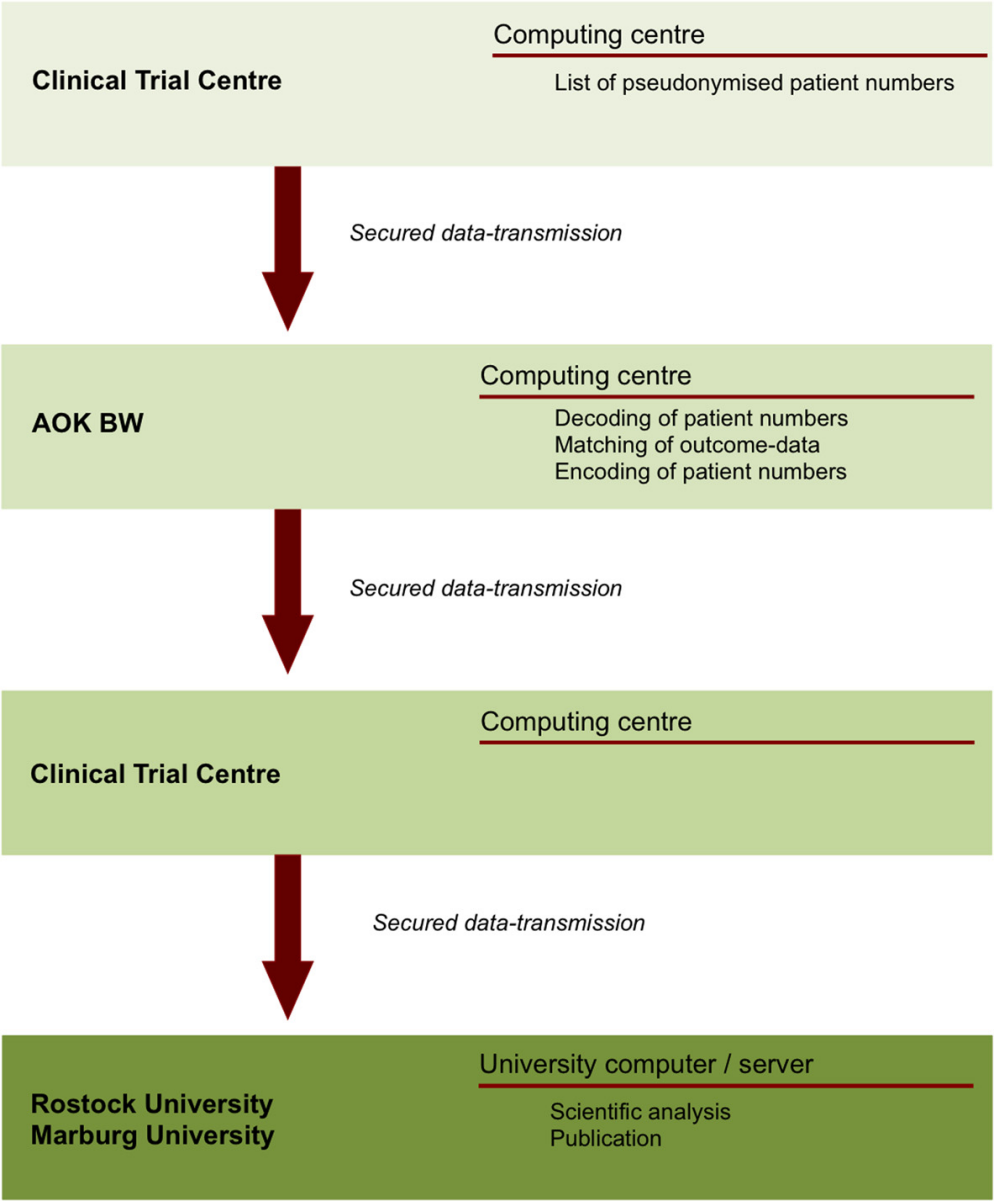
Data-matching and pseudonymisation steps.

In order to obtain information on outcome and treatments for participating patients, the AOK BW central database will regularly be queried. The resulting data will be provided to the academic departments at the universities of Marburg and Rostock via CTC North with all the precautions regarding confidentiality described above. These data will also include diagnostic codes, such as cardiovascular events, and relevant vascular procedures, such as percutaneous coronary interventions or coronary bypass surgery (see Figure 2).

### Data analysis

The main purpose of the analysis is the validation and adaptation of the risk algorithm presently implemented in the **arriba**-software [20]. The current algorithm is based on the Framingham cohort and is calibrated to German populations. Exposure variables are limited to the set currently recorded by the **arriba**-software (see Table 1). As a result of our study we will be able to adjust their weights (coefficients) as well as the intercept (base risk), possibly identify predictors that contribute little and can thus be omitted, and specify interactions between predictors.

We will use a stepwise procedure to analyze the data, going from the mere descriptive analysis to the univariate- and multivariate analysis, using the appropriate tools in each step.

Among the predictors to be entered into the multivariate model are time-independent variables, such as sex or previous cardiovascular disease, but also time-dependent factors, such as medication modifying cardiovascular risk. For pragmatic reasons, cholesterol and blood pressure levels will be regarded as time-independent since they are either relatively stable characteristics (LDL-/HDL-cholesterol) and/or cannot be measured over time within the current study design (blood pressure, smoking status). However, data on risk-modifying medication (e.g. statins, blood-pressure lowering drugs) can be retrieved from the AOK BW and be entered into the model. Treatment is likely to change over time but this can be accommodated in the statistical model.

To adjust for the confounders mentioned above, an extension of the Cox Proportional Hazards Model for time-dependent variables will be used as suggested by Kleinbaum [21]. To simulate a therapy naïve cohort, drug treatments will be adjusted for. The statistical analysis will be performed by the universities of Rostock und Marburg with support of the Department of Medical Biometry and Epidemiology, Hamburg.

### Sample size

In the main analysis, we aim to validate and re-calibrate the risk algorithm implemented in the **arriba**-software for primary prevention. To quantify the impact of a predictor, at least 10 events are needed [22]. If we assume patients in our cohort to have an average risk of a cardiovascular event of 5% in 10 years, the study of 9 predictors (see Table 1) would require a sample of at least 1,800 patients. However, further analyses will require a higher sample size. To validate the risk algorithm within the subgroup of patients with a known coronary heart disease a total sample size of 3,000 patients would be required if we assume that the percentage of this subgroup is 10% of the total sample and that the average risk of a cardiovascular event within this group is 30% in 10 years. Adjusting for the risk-modifying effect of treatment will raise the number of covariates to be entered in the models and the sample size accordingly. Within the first two years, 466 GPs have recruited about 4,100 patients. Considering a total time of enrolment of 4 years, the resulting sample size will meet our requirements with a sufficient safety margin.

### Ethics and funding

The study complies with the Declaration of Helsinki. The research protocol was approved by the ethics committee at the University of Marburg (ID 159/11). All patients who participate in the HzV AOK BW are asked to give their written consent for personal data to be used for research purposes.

A grant provided by the AOK allows a five years’ study duration. However, funding for an extended study period will be sought.

## Discussion

The main aim of the cohort study outlined here is the validation and adaptation of a cardiovascular risk algorithm.

Cardiovascular risk algorithms have repeatedly been shown to have limited predicting power when tested on independent samples [10,23]. There are many reasons for this unsatisfactory state, among these biological variability, demographic, social, and cultural differences between derivation and validation cohorts, comorbidities, and intervention by health care providers outside the study protocol, to name just a few.

Our study does address three of these in an innovative way:

Simulation of a therapy-naïve cohort: The main goal of the **arriba** risk calculator is the unbiased information of individual patients in primary care practice. For individuals considering long-term interventions such as lipid or blood-pressure lowering therapy, predictive evidence should be based on cohorts without interventions modifying cardiovascular risk. However, this is unfeasible since most subjects in the cohort will be treated by their health care provider once a single risk factor or their global cardiovascular risk is found to be elevated. By accommodating drug therapy during follow-up we will be able to simulate a therapy-naïve cohort and thus provide risk estimates not biased by pharmacological interventions. To our knowledge this has not been attempted before.

Match between research and application setting: Previously developed cardiovascular risk algorithms were derived from geographically or occupationally defined populations. This puts a limit on their generalizability regarding counseling of individual patients in primary care. Our study sample, however, is drawn from exactly the population for which the algorithm will be used once the study is completed.

Inclusion of patients with known cardiovascular disease: Previously published risk algorithms were derived from populations free of overt cardiovascular disease. They can thus not be used to inform decision-making for secondary prevention. Against the background of aging populations with high prevalence of cardiovascular disease, this is a serious limitation. As a result of our study, a risk calculator applicable to this group will be available.

The automatic **arriba**-data-collection of patients who are enrolled in the GP centered health care contract (HzV) is an elegant way to obtain relevant data. Patients participating in the HzV tend to be slightly older and suffer from a higher morbidity burden when compared with those AOK insured, who are not enrolled in the HzV [24]. With the study design presented here we can describe the characteristics of each individual of our cohort and so will be able to adjust for possible confounders.

Selection bias may arise from selective use of **arriba** by participating GPs. As a result, certain groups would be underrepresented in our study sample because their GPs are not interested in their **arriba** risk. This would apply to patients at obviously very high or very low risk. However, we are confident that, given our sample size, these groups will still be sufficiently represented in our study. Moreover, educational measures have been implemented to encourage GPs using **arriba** in a broad range of patients. If on the other hand GPs use the decision tool **arriba** in a ‘selective’ way, then this is obviously the population they feel to be in need of the information provided by the software. As a result, a sample specifically adapted to the needs of future users would be assembled.

We will use administrative data of the AOK BW to assess relevant medication as well as cardiovascular events. These data can be expected to be highly valid since they are based on claims by pharmacies, hospitals and physicians [25]. An important data source will be the hospital claims data set. In Germany a diagnosis related groups (DRG) system is used for reimbursement of hospital services. In the German DRG system, not only the main diagnosis but also secondary diagnoses and main procedures are coded and used to determine the final DRG [26].

Our study design is not without limitations. Smoking status will only be evaluated at baseline, we will not be able to take changes in smoking habits during follow-up as well as other behavioral risk factors into account. The set of risk factors (predictors) is limited to those currently included in the **arriba** risk calculator.

We are aware that in order to adapt and validate a ten-year cardiovascular risk calculator an extended follow-up period will be needed. Evidence based on shorter follow-up periods will be only preliminary. We therefore aim at longer follow-up than the five-year period that we have obtained funding for so far.

Despite these limitations, the study presented here provides an unusual opportunity to improve cardiovascular risk prediction for primary care. Simulation of a therapy naïve cohort, the exact match of research and application setting, a robust administrative database, and, finally, the inclusion of patients with known cardiovascular disease make this study unique in comparison with previous adaptations of cardiovascular risk algorithms.

## Competing interests

AA and NDB are chairmen of a non-profit company providing decision support software to health care organisations and individual providers. The **arriba** software is available free of charge from ‘http://www.arriba-hausarzt.de’. KK is leader of the department prevention at the Federal Association of the AOK, KT is project manager at the AOK Baden-Württemberg. The remaining authors declare that they have no competing interests.

## Authors’ contribution

AA and NDB conceived the study. All authors contributed to the planning of the study. AD, SHC, and JH drafted the manuscript. All authors commented on the draft and approved the final version of the manuscript.

## Funding

This study is funded by the AOK Bundesverband (German Federal Association of Local Health Insurance Funds) and AOK Baden-Württemberg (Baden-Württemberg Statutory Health Insurance Fund).

## Pre-publication history

The pre-publication history for this paper can be accessed here:

http://www.biomedcentral.com/1471-2296/14/13/prepub
